# A Simple Maneuver to Improve Catheter Stability at the Posterior Right Pulmonary Vein in a Small Left Atrium

**DOI:** 10.1002/joa3.70372

**Published:** 2026-05-29

**Authors:** Yosuke Nakatani, Shuntaro Tamura, Hiroshi Hasegawa, Ryutaro Iwai, Hideki Ishii

**Affiliations:** ^1^ Division of Non‐Pharmacological Management of Cardiac Arrhythmias Gunma University Graduate School of Medicine Maebashi Japan; ^2^ Department of Cardiovascular Medicine Gunma University Graduate School of Medicine Maebashi Japan

**Keywords:** catheter ablation, left atrium, pulmonary vein isolation, pulsed field ablation

## Abstract

A novel sheath–catheter maneuver enables stable and flush Varipulse ring positioning at the posterior antrum of the right pulmonary veins in anatomically constrained left atria, where conventional techniques may fail.

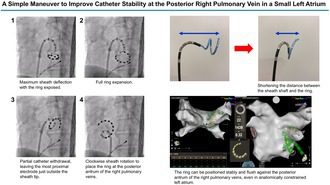

The Varipulse pulsed field ablation (PFA) system enables durable pulmonary vein (PV) isolation using a multielectrode ring catheter equipped with a tissue proximity indicator. Because the system does not employ guidewire anchoring, catheter stability depends largely on coordinated manipulation of the catheter and the steerable sheath. Achieving stable contact along the posterior antrum of the right PVs can be challenging in patients with a small left atrium (LA), particularly when the posterior LA wall is compressed by the vertebrae. In such anatomies, the limited distance between the transseptal puncture site and the posterior wall hampers flush alignment of the Varipulse ring against the posterior antrum with conventional techniques.

We present a simple and reproducible maneuver to improve catheter stability under these conditions. After advancing the Varipulse catheter through a deflectable sheath into the LA, the following steps are performed (Figure 1 and Video S1): (1) the steerable sheath is maximally deflected while the Varipulse ring remains exposed beyond the sheath tip; (2) the ring is fully expanded to its largest diameter; (3) the catheter is partially withdrawn until the most proximal electrode of the expanded ring is positioned just outside the sheath tip; and (4) the sheath is rotated clockwise to position the ring against the posterior antrum of the right PVs.

**FIGURE 1:**
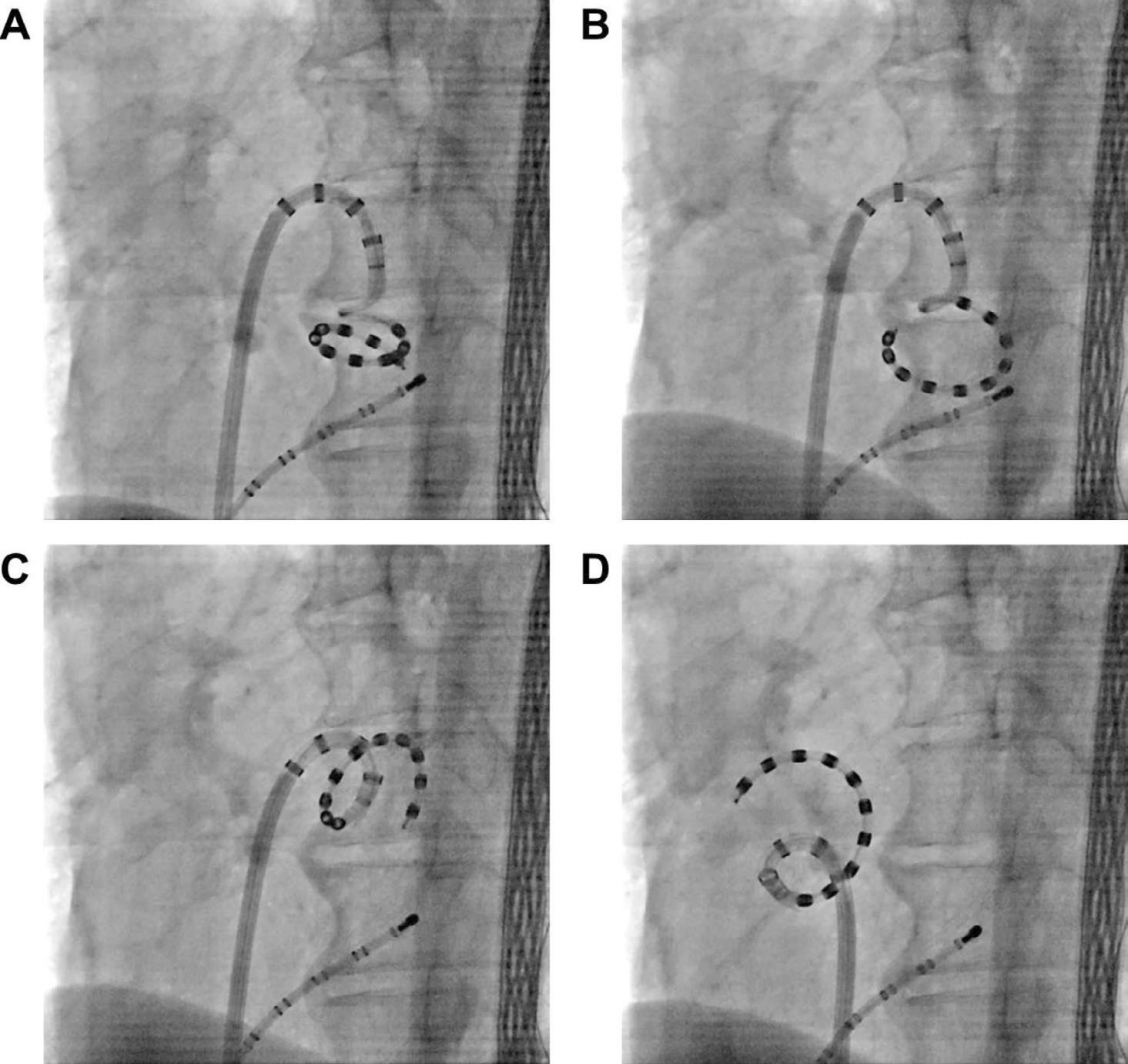
Stepwise maneuver for positioning the Varipulse ring at the posterior antrum of the right pulmonary veins (PVs). All images are fluoroscopic views in the left anterior oblique 45° projection. (A) Maximum sheath deflection with the ring exposed. (B) Full ring expansion. (C) Partial catheter withdrawal, leaving the most proximal electrode just outside the sheath tip. (D) Clockwise sheath rotation to place the ring at the posterior antrum of the right PVs. The corresponding video is provided as Video S1.

This maneuver aligns the Varipulse ring nearly parallel to the sheath shaft, thereby shortening the distance between the sheath shaft and the ring and reducing the rotational radius during sheath rotation (Figure 2). With this tightly coupled configuration, the ring can be positioned stably and flush against the posterior antrum of the right PVs, even in anatomically constrained LA (Figure 3 and Video S2). In addition, gentle caudal traction on the sheath facilitates stable placement at the inferior aspect of the right inferior PV, a region in which catheter stability is often suboptimal during PFA.

**FIGURE 2:**
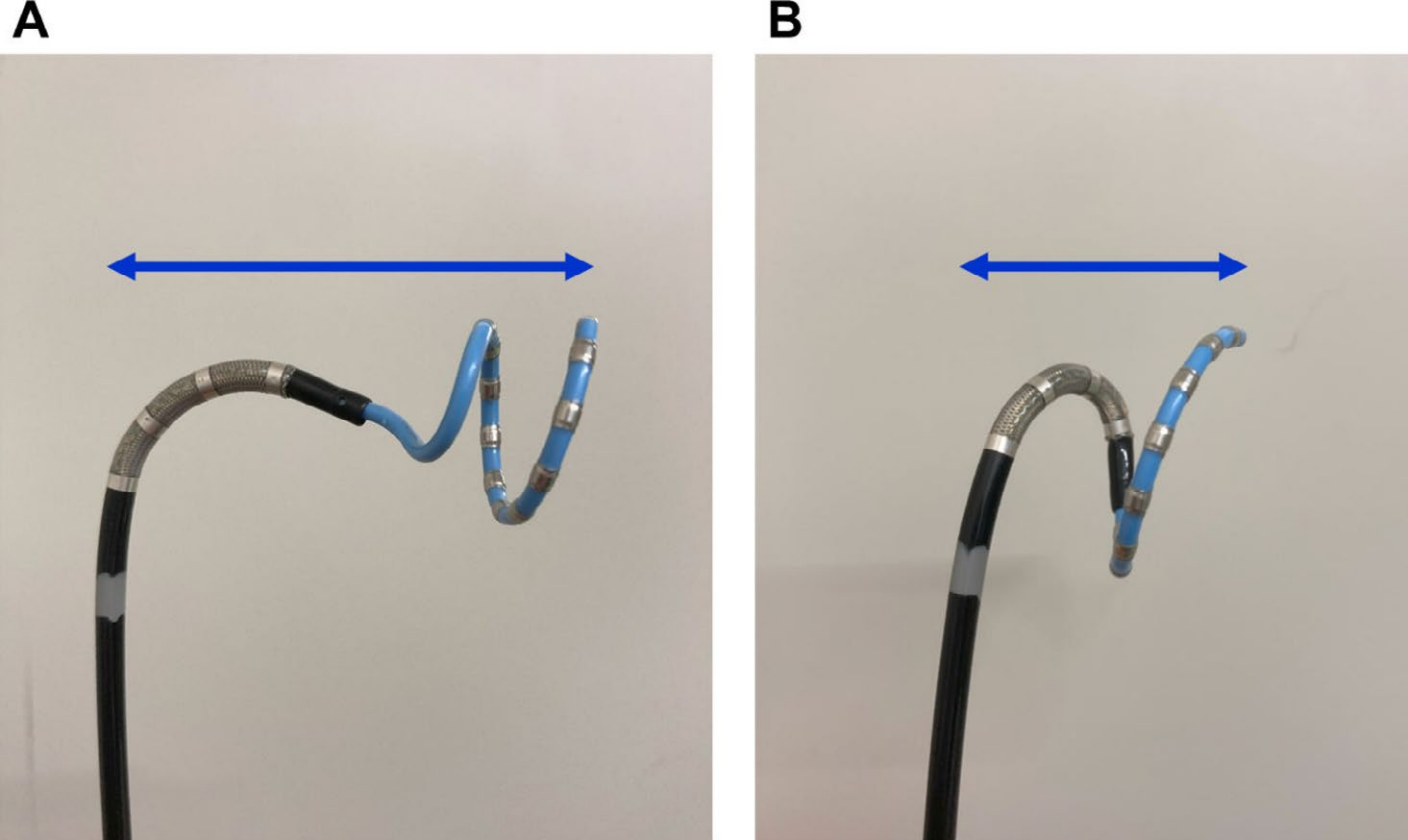
Comparison of catheter configuration between the conventional technique and the technique described in this report. (A) Conventional catheter configuration. (B) Catheter configuration using this technique. The distance between the sheath shaft and the ring catheter (blue arrow) is shorter than that in the conventional technique.

**FIGURE 3:**
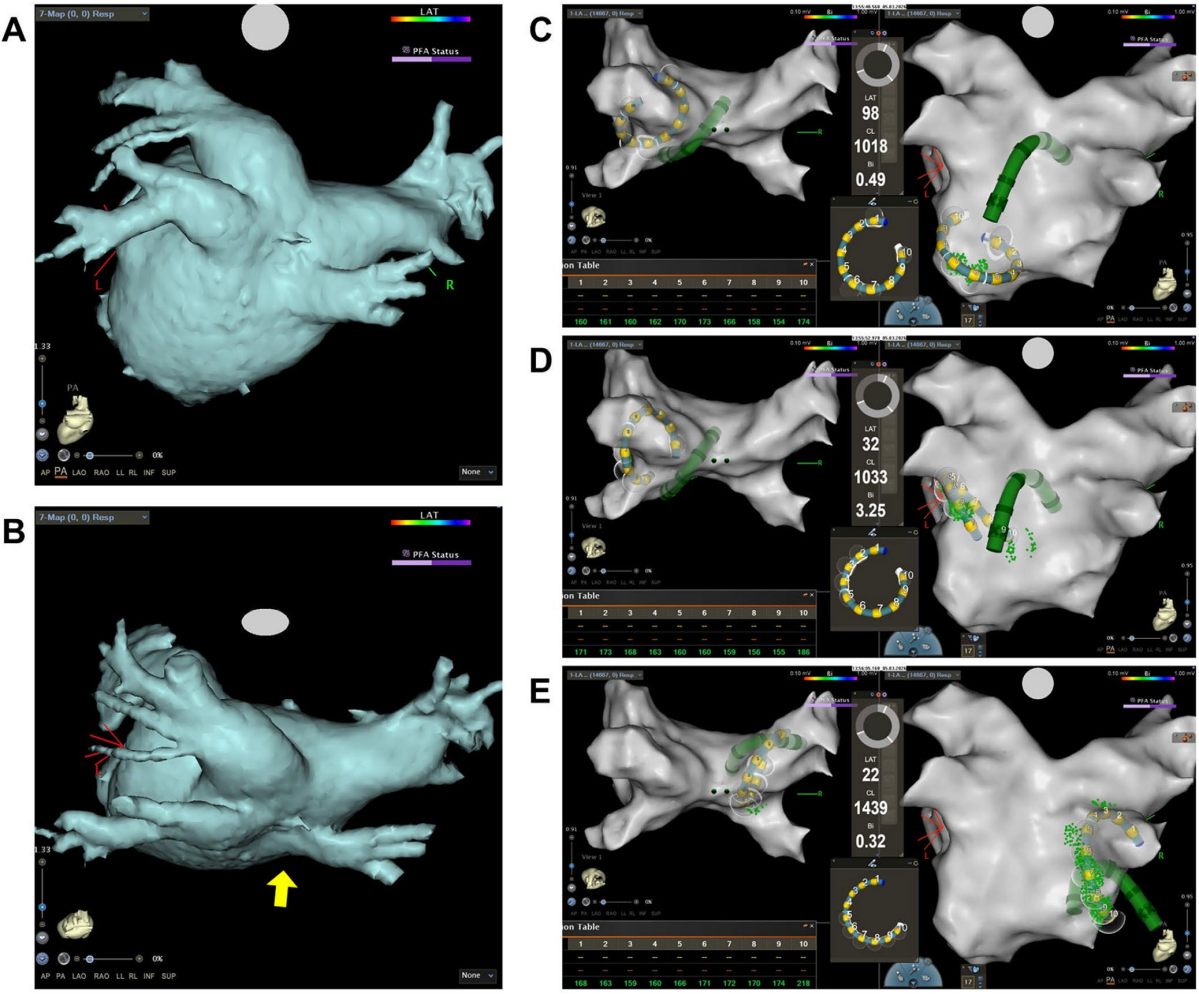
Representative case demonstrating the technique described in this report in an anatomically constrained left atrium. (A, B) Three‐dimensional reconstructed computed tomography images of the left atrium: (A) posterior view and (B) superior view, demonstrating compression of the posterior wall of the left atrium by the vertebra (yellow arrow). The left atrial volume was 47 mL. (C–E) Representative catheter manipulation on three‐dimensional mapping: (C) sheath deflection with the ring deployed and fully expanded; (D) catheter withdrawal resulting in near‐parallel alignment of the Varipulse ring with the sheath shaft; and (E) clockwise sheath rotation achieving stable and flush contact of the ring at the posterior antrum of the right pulmonary veins. The corresponding video is provided as Video S2.

In contrast, in patients with a larger LA, this configuration may limit posterior reach of the ring, and conventional positioning techniques remain preferable. With the conventional technique, the minimum distance between the sheath shaft and the ring is approximately 50 mm, whereas this distance is reduced to about 25 mm with our maneuver. Accordingly, our maneuver is particularly suitable in cases where the distance from the fossa ovalis—that is, the presumed transseptal puncture site—to the posterior antrum of the right PVs is ≤ 25 mm on preprocedural computed tomography. In clinical practice, it is more practical to switch to our maneuver intraoperatively when stable contact with the posterior antrum of the right PVs cannot be achieved using the conventional technique. In our experience, in patients with a small LA in whom the conventional technique was unsuccessful, our maneuver was effective in all cases.

Overall, this simple sheath–catheter manipulation effectively enhances catheter stability at the posterior antrum of the right PVs in anatomically limited LA and may broaden the range of anatomies suitable for Varipulse‐based PFA.

## Funding

The authors have nothing to report.

## Consent

The authors have nothing to report.

## Conflicts of Interest

Y.N. is affiliated with an Endowed Department funded by DVx Inc., Medtronic Japan, Biotronik Japan, Boston Scientific Japan, and Japan Lifeline.

## Supporting information


**Video S1:** Stepwise maneuver for positioning the Varipulse ring at the posterior antrum of the right pulmonary veins. This video corresponds to Figure 1.

**Video S2:** Representative case demonstrating the technique described in this report in an anatomically constrained left atrium. This video corresponds to Figure 3.

## Data Availability

The data that support the findings of this study are available from the corresponding author upon reasonable request.

